# Roles of piRNAs in transposon and pseudogene regulation of germline mRNAs and lncRNAs

**DOI:** 10.1186/s13059-020-02221-x

**Published:** 2021-01-08

**Authors:** Chen Wang, Haifan Lin

**Affiliations:** 1 Shanghai Institute for Advanced Immunochemical Studies, ShanghaiTech University, Shanghai, 201210 China; 2 Yale Stem Cell Center and Department of Cell Biology, Yale University School of Medicine, New Haven, CT 06519 USA

## Abstract

PIWI proteins, a subfamily of PAZ/PIWI Domain family RNA-binding proteins, are best known for their function in silencing transposons and germline development by partnering with small noncoding RNAs called PIWI-interacting RNAs (piRNAs). However, recent studies have revealed multifaceted roles of the PIWI-piRNA pathway in regulating the expression of other major classes of RNAs in germ cells. In this review, we summarize how PIWI proteins and piRNAs regulate the expression of many disparate RNAs, describing a highly complex global genomic regulatory relationship at the RNA level through which piRNAs functionally connect all major constituents of the genome in the germline.

## Introduction

**Fig. 1:**
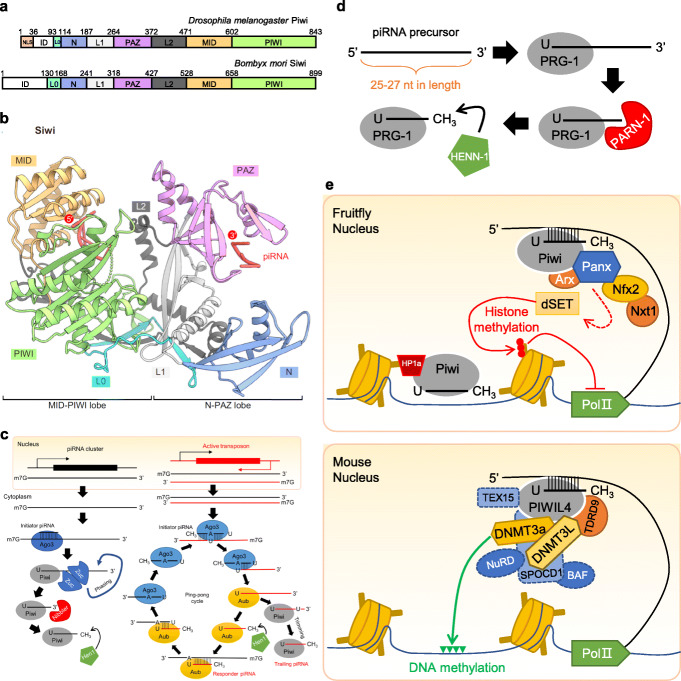
Overview of the PIWI-piRNA pathway. **a** Structures of the fruitfly Piwi and silkworm Siwi [2, 3]. Piwi and Siwi are highly conserved and shared eight regions that form four common domains. The N-terminal domain, PAZ domain, MID domain, and PIWI domain and interspersed by L0, L1, and L2 sequences. The nuclear localization signal (NLS) in the N domain of Piwi enables the nuclear localization. Siwi does not contain an NLS and is a cytoplasmic protein. **b** The first 3D structure of a Piwi protein-Siwi by Matsumoto et al. [2]. Siwi is divided into two lobes. L0, N, L1, PAZ, and L2 domains form the N-PAZ lobe, whereas L0, L2, MID, and PIWI domains form the MID-PIWI lobe. These two lobes give rise to a nucleic acid-binding channel. **c** Biogenesis of piRNAs in *Drosophila*. piRNA precursor is transcribed from piRNA clusters and exported from nucleus. 5′ end monophosphorylated piRNA precursors are bond by Piwi protein loaded with initiator piRNAs and processed into pre-piRNA and pre-pre-piRNA. Pre-pre-piRNAs are phased by Zucchini endonuclease (Zuc) [4–6] while pre-piRNAs are trimmed by Nibbler to produce mature piRNAs [7, 8]. Finally, the 3′ end of piRNAs are 2′O-methylated by the Hen1 methylase [9–12]. After 2-O-methylation, the mature piRNA-Piwi complex further initiates ping-pong piRNA biogenesis facilitated by the other two Piwi proteins Ago3 and Aub [13, 14]. **d** Biogenesis of piRNAs in *C. elegans* [15]. piRNA precursors in *C. elegans* are short RNAs with a length of 25–27 nt. The processed piRNA precursors were also trimmed and 2′O-methylated by respective nucleases to generate mature piRNAs. **e** Prevalent models for Piwi-mediated transcriptional silencing of transposons. In the nucleus of fruitfly (upper panel), the Piwi-piRNA complex binds to a nascent transposon transcript and recruits Panoramix (Panx) and Asterix (Arx) mediator complex to the vicinity of the target chromatin region. Panoramix further interacts with Nxf2 and Nxt1 and which recruits histone methytransferase, dSetDB1(dSET) to methylate the Lysine residue at the 9th position of histone 3, which establishes a repressive chromatin state to suppress transposon expression [16–22]. In mice (lower panel), piRNA-loaded MIWI2 associates with TDRD9, DNMT3L, DNMT3a, and DNMT3a2 in transfected 293 T cells. The complex is guided by piRNA and deposits DNA methylation via DNMT3a2 [23]. However, the latest reports indicated that SPOCD1 links MIWI2 to Dnmt3a and Dnmat3L and the complex also contain TEX15 [24, 25]. Therefore, how exactly these proteins interact with each other remain unknown

PIWI proteins represent one of the two subfamilies of the PAZ/PIWI Domain (PPD) protein family, with the other subfamily termed Ago proteins. All PPD family proteins contain a variable N-terminal domain followed by a highly conserved PAZ domain, which together with the MID domain binds a small RNA [1, 2] (Fig. 1a, b). The C-terminal PIWI domain resembles RNase H and in some PPD family proteins are capable of cleaving target RNAs [26–28]. Ago subfamily proteins are present in most types of cells. They bind to microRNAs (miRNAs) and small interfering RNAs (siRNAs), both of which are 21-nucleotide in length and are produced from double-stranded precursors via a Dicer-dependent process [29]. In contrast, PIWI subfamily proteins are mostly expressed in the germline, even though in most arthropod species, PIWI proteins are expressed in the soma as well [30–32]. PIWI proteins bind to piRNAs that are generally 24–32 nucleotides in length and are also enriched in the germline [33–36].

PiRNAs in most organisms are processed from long single-stranded precursors in a Dicer-independent manner (Fig. 1c), except for *C. elegans*, in which piRNAs (21 U-RNAs) are produced from short, single-stranded precursors (~ 26 nt capped transcripts) in a Dicer-independent manner [15, 37] (Fig. 1d). The long single-stranded precursors RNAs were transcribed from loci termed piRNA clusters in the genome and processed through ping-pong cycles that post-transcriptionally amplifies piRNAs with overlapping complementarity by accelerating their production from precursors by the alternative action of their associated PIWI proteins as follows [13, 26, 33–36] (Fig. 1c). The transcribed long precursors are initially processed into 5′ end monophosphorylated RNAs by PIWI protein loaded with initiator piRNAs. The processed precursors (pre-pre-piRNAs) are further cleaved by PIWI proteins to generate responder pre-piRNAs from the 5′ ends of pre-pre-piRNAs. These pre-piRNAs are trimmed by exonucleases to generate functional piRNAs [38–41] and give rises to mature responder piRNAs that recognize the complementary strands of long precursors and initiated the ping-pong cycles. Meanwhile, 5′ end monophosphorylated precursor RNAs are fragmented into a string of phased trailing pre-RNAs by PIWI and PIWI-coupled-proteins on the mitochondrial outer membrane [4–6]. Importantly, piRNAs are symbolically 2′-O-methytlated at the 3′ end by S-adenosylmethionine-dependent methyltransferases [9–12]. Perhaps the two best-known functions of PIWI proteins and piRNAs are transposon silencing and fertility [42]. However, many recent studies have started to reveal a much broader role of the PIWI-piRNA pathway in meditating the regulation of major constituents of the genome, which is the focus of this review.

## The function of PIWI proteins and piRNAs in transposon silencing and fertility: an update

Before reviewing the new functions of the PIWI-piRNA pathway, here we provide an update on its function in transposon silencing and fertility, which also serves as needed background information of the rest of the review. Transposon silencing has been widely regarded as a requirement for fertility. However, this relationship has not been supported by definitive evidence. In contrast, these two functions are separable at least in *Drosophila* [43] and mice [44].

Transposon silencing is achieved by repression the expression of retrotransposon RNAs at both transcriptional and posttranscriptional levels [45]. Transcriptional silencing is mediated by nuclear PIWI proteins such as PIWI in *Drosophila* and MIWI2 (a.k.a. PIWIL4) in mice, whereas posttranscriptional silencing is mediated by cytoplasmic PIWI proteins such as Aubergine (Aub) and Ago3 in *Drosophila* or MIWI (a.k.a. PIWIL1) and MILI (a.k.a. PIWIL2) in mice. A prevalent model of transcriptional silencing in *Drosophila* is that the PIWI-piRNA complex binds to a nascent transposon transcript (Fig. 1e upper panel). The complex interacts with mediator proteins Asterix and Panoramix (a.k.a. Silencio). Furthermore, Panoramix interacts with Nxf2 (Nuclear Export Factor 2) and Nxt1 (Nuclear Transport Factor 2 Like Export Factor 1) which recruit a histone methytransferase, dSetDB1 (a.k.a. Eggless), to methylate Lysine 9 residue in histone 3 (H3K9). This promotes the repressive chromatin state [16–22, 46–48]. Alternative models such as the *Drosophila* Piwi protein directly recruiting Heterochromatin Protein 1a to initiate heterochromatinization or recruiting the linker histone H1 to induce repressed chromatin state have also been proposed [49–51]. The above three models do not mutually exclude each other and might indeed co-exist in the cell. They share two common features: (1) transcriptional repression requires the specific binding of a PIWI-piRNA complex to a nascent RNA at the target site to recruit epigenetic/chromatin factors but does not require the PIWI slicer activity [52, 53]; (2) transcriptional repression occurs by modifying chromatin structure.

In mice, piRNA-dependent transcriptional silencing in the germline is also achieved by methylation of DNA through a recruitment scheme that is less known but similar to that in *Drosophila* [23, 54–60] (Fig. 1e, lower panel). For example, Tudor Domain-Containing 9 (TDRD9) complexes with MIWI2 and suppresses transposons in a piRNA-dependent manner [61]. MIWI2 is guided by piRNA to the nascent transcript of specific genomic region to mediate the methylation of the target DNA [60]. MIWI2 in turn appears to directly recruit with DNMT3A, DNMT3A2, AND DNMT3L, and TDRD9 to achieve methylation, since MIWI2 seems to directly interact with these proteins in co-expression and immunoprecipitation assay in a 293 T cell [23]. However, a most recent report indicates that a nuclear protein, SPOCD1, interacts with MIWI2 as well as with DMNT3A, DNMT3L, and components of NURD and BAF chromatin remodeling complexes during de novo DNA methylation [24]. Among the DNA methylases, DNMT3C functions as one of the key methyltransferases that protects male germ cells from transposon activity [62]. Another nuclear protein, TEX15, also interacts with MIWI2 in the process [25]. A separate report indicates that TEX15 interacts with MILI, and the genome of *Tex15* mutant is hypomethylated similar to *Mili* and *Dnmt3c* but not *Miwi2* mutants [63]. Therefore, the above have revealed some of the key players in the PIWI-piRNA-mediated mechanism in the de novo DNA methylation, but substantial work is still needed to delineate their exact interactions and relative contributions to DNA methylation.

In addition, a recent study showed that the Ubiquitin-like, Containing PHD and RING finger Domains 1 (UHRF1) protein may interact with MIWI and MILI to deposit DNA methylation or to further recruit PRMT5 to methylate arginine of histones [64]. This study demonstrated the requirement of UHRF1 in histone and DNA methylation. However, the co-immunoprecipitation data did not convincingly show the UHRF1-MIWI/MILI interaction. In addition, MIWI and MILI are in the cytoplasm yet UHRF1 is in the nucleus. Thus, the nature of MIWI/MILI interaction with UHRF1 remains to be further explored. Finally, the zinc finger protein MORC1 promotes DNA methylation of transposons but not protein-coding genes in male germ cells during the period of global de novo methylation [65].Whether/how these proteins work together to methylate the genome of male germ cells awaits further investigation.

Post-transcriptional silencing occurs in the cytoplasm, where a PIWI-piRNA complex binds to a piRNA-complementary transposon RNAs and cleaves the RNA by the slicer activity of the PIWI protein (Fig. 1c). This silencing meanwhile fulfills piRNA biogenesis. Both transcriptional and post-transcriptional silencing of transposons by the piRNA pathway has been well-reviewed (e.g., [66]); thus, this topic will not be further covered in our review.

In contrast to transposon silencing, little is known about whether/how PIWI proteins and piRNAs regulate gene expression and other major types of RNAs in the germline. In this review, we summarize recent research on this less-investigated topic. Recent progress has led to surprising discoveries that transposons actively regulate the expression of mRNAs and long noncoding RNAs (lncRNAs), mediated by the PIWI-piRNA pathway. Moreover, pseudogenes also regulate the expression of their cognate mRNAs, also mediated by the PIWI-piRNA pathway. Finally, the PIWI-piRNA pathway even regulates the expression of (sub-) telomeric and (peri-) centromeric transcripts. These surprising findings have begun to reveal genome-wide functions of the PIWI-piRNA pathway in functionally links the major classes of genomic sequences and protein-coding genes at the RNA level.

## Regulation of mRNAs by transposons

**Fig. 2:**
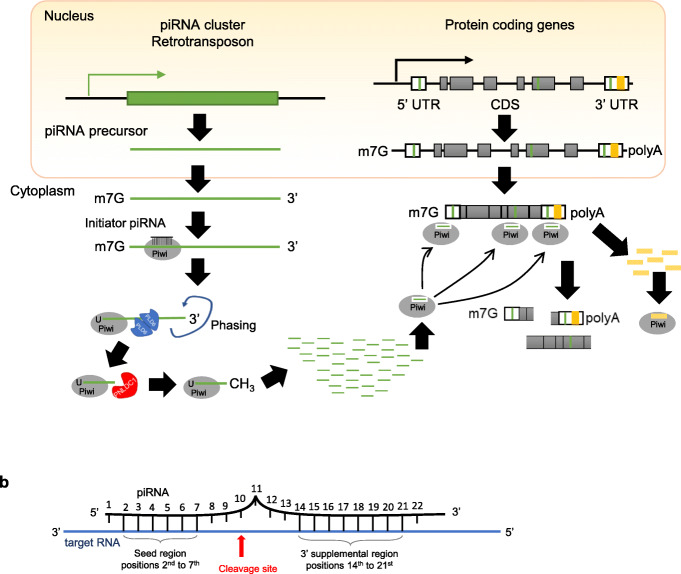
piRNA mediates mRNA regulation by transposons. **a** piRNA precursors are transcribed from piRNA clusters that include retrotransposon sequences (the green box). Mature piRNAs (short green lines) associated with Piwi proteins guide the Piwi-piRNA complex to complementary transposon sequences predominately in 3′ UTR and occasionally to 5′ UTR or CDS of specific mRNAs [67–69]. The targeted mRNAs are degraded through Piwi-slicing and other mechanisms (see Fig. 3). Alternatively, piRNAs are generated from mRNA that contains transposon sequences (the orange box) in the 3′ UTR. These piRNAs (orange short lines) in turn target corresponding mRNAs and mediate their degradation [73–75]. **b** The piRNA-target RNA pairing rule as exemplified by *C. elegans* studies. Other organisms appear to follow a similar rule

A recently discovered function of the piRNA pathway in the cytoplasm is to mediate the regulation of mRNAs by transposons (Fig. 2). This discovery came as a surprise at multiple levels—from realizing that many mRNAs contain transposon sequences to learning that transposons can use these sequences as Trojan’s horses to regulate target mRNAs, and to revealing the essential role of PIWI proteins and piRNAs in this regulation.

### Many mRNAs in mammals contain transposon sequences in the 3′ untranslated region

The first hint of transposons regulation towards mRNAs came from observations that some expressed retrotransposon sequences, especially short interspersed nuclear element (SINE) and long interspersed nuclear element (LINE) families, mapped to the 3′ untranslated regions (UTRs) of mRNAs in the mammalian genome [67, 68] (Fig. 2). For example, 27.7% of mouse and 28.5% of human mRNAs contain at least one retrotransposon fragment, often a SINE, that is predominantly in the 3′ UTR of mRNAs (Table 1), while a few others are in 5’UTR and even fewer are in protein-coding sequences (CDS) [68]. In *Drosophila*, densities of putative piRNA target sites in both the 5′ UTR and especially the 3′ UTR regions are also higher than that in coding sequences [69], even though this bias is not as pronounced as in mammalian genomes. The paucity of the transposon insertions in CDS likely results from evolutionary selection, because an insertion in CDS disrupts a protein’s sequence and often its function, and thus, it is eliminated from the gene pool. However, there are frequent instances in which retrotransposon insertions reduce host mRNA expression [67, 68], indicating a potential role of the inserted transposon sequence in regulating mRNA expression.

**Table 1:** Classification of retrotransposon sequence in mRNA 3′ UTR

Retrotransposon [67]	Human 3’ UTR	Mouse 3’ UTR
DNA	6.6%	2.9%
LINE.L1	4.7%	2.8%
LINE.L2	4.2%	2.5%
LTR.ERV1	6.4%	2.9%
LTR.ERVK	0.7%	2.6%
LTR.ERVL	4.5%	4.3%
LTR.MALR	5.2%	3.8%
SINE.Alu	5.7%	4.7%
SINE.B2	N.A.	5.0%
SINE.B4	N.A.	4.8%
SINE.MIR	2.7%	6.3%

### Transposon sequences in mRNAs are Trojan’s horses that lead to mRNA degradation

Recent studies indicate that transposon sequences in mRNAs are the targeting sites of transposon-derived piRNAs that degrade these mRNAs [68] (Fig. 2a). MIWI promotes the degradation of the mRNA from a mCherry reporter containing a B1 or B2 SINE sequence at its 3′ UTR [68]. This RNA-degradation function depends on its slicer function. RNA immunoprecipitation (RIP) against MIWI and MILI followed by RT-PCR, as well as high-throughput sequencing of RNA isolated by crosslinking immunoprecipitation (HITS-CLIP) in the adult mouse testis showed that MIWI and MILI directly bind to mRNAs [76, 77]. Additionally, MIWI binds to mRNAs as a mRNA ribonucleoprotein complex in the testis [77, 78]. MIWI CLIP-seq indicates that piRNAs preferably bind to the 3′ UTR of mRNAs in mouse elongating spermatids [72, 76]. Moreover, a group of meiotic mRNAs were significantly upregulated in *Miwi*^*−/−*^ mice round spermatids, and the degradation of meiotic mRNA exhibited piRNA generation signatures [68, 79]. All these findings indicate that PIWI proteins and their associated piRNAs might be directly involved in regulating the stability of target mRNAs.

The involvement of transposon-derived piRNAs in regulating their target mRNAs is further supported by experiments that investigate the effect of deleting piRNAs or their target sequences on mRNA stability. In mouse late spermatocytes deficient in *Miwi* or *Mov10l1*, another gene involved in piRNA biogenesis, piRNA biogenesis is deficient, and the expression levels of a large number of protein-coding genes are altered [68]. In addition, depletion of the retrotransposon sequences in either the 3′ UTR of *prelid1* or the piRNA cluster that produces the targeting piRNAs results in a six-fold up-regulation of the *prelid1* mRNA level in late spermatocytes. Furthermore, this MIWI- and MOV10L1-mediated regulation of mRNAs requires the slicer activity of MIWI [68, 80]. Most recently, it was reported that deleting a specific piRNA cluster, *pi6*, led to upregulating of mRNAs related to sperm function, in addition to affecting piRNA-piRNA precursor interactions [81]. This provides an additional example for piRNA function in regulating mRNAs and in spermatogenesis.

The suppression of retrotransposon-containing mRNAs in mouse primordial ovarian follicles also requires MILI and other piRNA pathway components such as mouse Vasa homolog (MVH) and TDRD9 [82]. In *Drosophila*, piRNAs suppress the *Stellate* repeat mRNA to ensure spermatogenesis, even though this regulation was initially reported as a siRNA-mediated mechanism [83]. These piRNAs, however, cannot repress the *Stellate* mRNA from closely related *Drosophila* species, thus introducing reproductive isolation [84]. All these observations demonstrate a direct role of PIWI proteins and their partner piRNAs in regulating mRNAs.

### PIWI-piRNAs targeting rules

The slicing of piRNA-targeted mRNA mostly occurs at the 10th position from the 5′ end of the targeting piRNAs [13, 68]. In-depth analysis of the cleavage site in the 3′ UTR of *Tdrd1* mRNA with respect to its targeting piRNAs reveal that most piRNAs are aligned antisense to the mRNA sequence with their 5′ nucleotide being 10 nucleotides away from the cleavage site, but several percent of piRNAs are aligned with their 5′ nucleotide 11–20 nucleotides away [68]. This could indicate that the cleavage site for these piRNA-complementary mRNAs is downstream of the 10th position of piRNAs.

Despite the relatively clear information on the cleavage site as supported by in vitro, in vivo, and structural analyses, the piRNA targeting rule is still elusive and increasing effort has been put into revealing this puzzle. In *C. elegans* and mice, piRNA binding to target RNA is sequence-dependent but not completely sequence-specific, with position 2–8 as the seed sequence and 14–22 nucleotides are also important for piRNA targeting [69, 72, 85, 86], reminiscent of the microRNA seed sequence for target recognition (Fig. 2b). Further attempts in ectopic expression of human piRNAs in mouse testes show that perfect matching in nucleotides 2–11 of piRNA is required for mRNA targeting [79]. In a different study with deep RNA sequencing that allowed up to four mismatches at random positions in a piRNA, all of the 172 mRNAs that were significantly upregulated in *Piwil1*^*−/−*^ testes contain transposon piRNA target sites in their 3′ UTR, with such a transposon sequence experimentally demonstrated to be responsible for MIWI-piRNA-mediated degradation [68]. Notably, these 172 mRNAs are quite different from the MIWI-target RNAs identified by Vourekas et al. (2016) using iCLIP. This likely reflects that iCLIP approach revealed stable binding of PIWI to target mRNAs that do not lead to mRNA degradation but possibly other regulatory effects, in contrast to RNA sequencing that revealed the degraded products of MIWI binding and cleavage of another set of target mRNAs (i.e., the mRNA degradome).

In *Drosophila*, mutating 1, 2, 3 nucleotides of a given piRNA in different positions did not abolish the binding ability of Piwi to its target nascent RNA at the genomic site, but reduce its binding affinity proportional to the number of mutations [50]. These observations also support the notion that piRNA targeting is sequence-dependent but not completely sequence-specific.

### mRNAs also produce piRNAs

The source of piRNAs is sometimes mRNAs. In *Drosophila* ovaries, transposons that are integrated in the 3′ UTR of actively transcribed genes induce piRNA production, which in-turn suppresses corresponding gene expression [73, 74] (Fig. 2a, right part). For example, in the *Drosophila* ovarian germline, proto-oncogene *c-Fos* is repressed post-transcriptionally in germline stem cells (GSCs) by Piwi protein. As a by-product of this repression, piRNAs are produced from the transposon elements in the 3′ UTR of *c-Fos* mRNA [74]. This repression is critical for GSC self-renewal and differentiation. In the *Drosophila* ovarian somatic cell (OSC) line, the 3′ UTR of *c-Fos* mRNA alone is sufficient to introduce gene suppression in a Piwi-dependent manner [74]. Similarly, in this OSC line, the 3′ UTR of a protein-coding gene *traffic jam* produces piRNAs to repress the expression of another protein-coding gene, *fasciclin III* [75]. However, in this case, the *traffic jam* piRNAs are not transposon-originated piRNAs.

### piRNAs can target the protein-coding sequence of mRNA for its degradation

A few publications have reported that piRNAs can also target the protein-coding sequence (CDS) of a mRNA to degrade the mRNA. For instance, in the *Drosophila* embryo, Aub directly binds to the CDS and the 3′ UTR of maternal mRNAs in a piRNA-dependent manner to facilitate the decay of these maternal mRNAs [87]. The Aub-piRNA complex is also associated with CCR4 in *Drosophila* GSCs and represses the expression of *Casitas B-cell lymphoma* (*Cbl*) mRNA [88]. This mRNA contains target sites for transposon-derived piRNA in both 5′ UTR and 3′ UTR, and these target sites overlap with Aub CLIP hits. In *Caenorhabditis*, a sex-chromosome-derived piRNA suppresses the expression of *xol-1* (*XO Lethal*), the master sex determination gene, via targeting the CDS region of the *xol-1* mRNA [89]. In the silkworm, a *Feminizer*-derived piRNA targets the CDS of the *Masculinizer* mRNA and determines the sex of the silkworm [90]. In human breast cancer cell lines, a piRNA called *piR-FTH1* shows an inverse correlation with the expression of *Fth1* (*ferritin heavy chain 1*) [91]. MILI and MIWI2 bind to *piR-FTH1* to repress the *Fth1* mRNA via targeting the CDS region of *Fth1* [91]. These observations from diverse and independent processes, each of which with biological significance, indicate that transposon sequences in CDS are equally effective in regulating mRNA stability. Thus, the paucity of piRNA target sites in the CDS is likely only due to their disruptive effect on protein-coding capacity but not a lower efficiency for piRNA-targeted degradation.

Finally, the insertion of transposon sequences into mRNAs as piRNA target sequences appears to be a dynamic process at least for certain cell types. The transposon landscape in cultured *Drosophila* ovarian somatic sheet (OSS) cells differs from that of cultured *Drosophila* OSCs [92], indicating that factors even as culture conditions may select for transposon insertions that are in favor of the regulation of mRNA expression that meets the needs of the given condition.

### Protein machinery involved in mRNA degradation by transposon-derived piRNAs

**Fig. 3:**
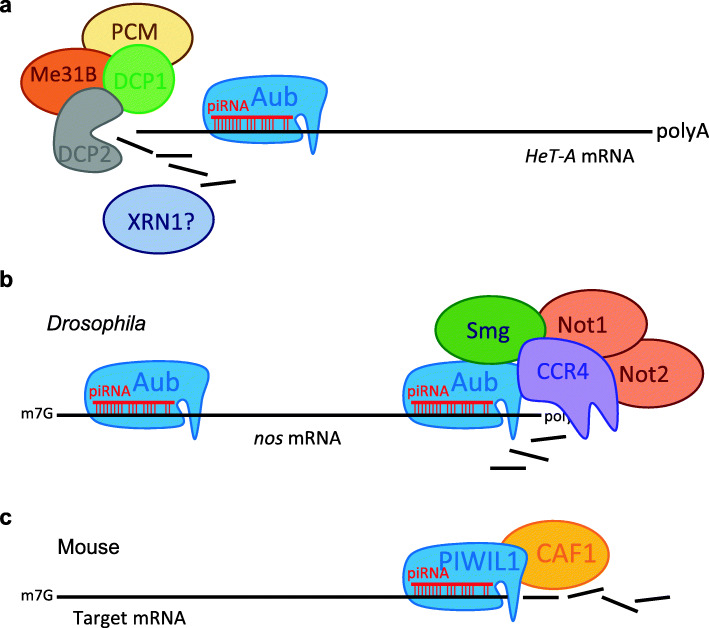
The piRNA-Piwi complex recruits canonical mRNA processing and localization machineries to regulate mRNAs. **a** In the *Drosophila* germline, the mRNA de-capping complex, which includes PCM, Me31B, DCP1, and DCP2 proteins, is recruited by the piRNA-Piwi complex to *HeT-A* mRNA to promote the decay of *HeT-A* mRNA [70]. **b** In *Drosophila* embryos*,* the piRNA-Piwi complex recruits the Smg protein and CCR4 complex to the 3′ UTR of *nos* mRNA to facilitate the degradation of *nos* mRNA [71]. **c** In the mouse testis, the piRNA-Piwi complex interacts with Caf1 bound to the 3′ UTRs of target mRNAs to execute mRNA degradation through the slicer activity of Piwi [72]

A few studies have further suggested that the PIWI-piRNA regulation of mRNAs involves canonical mRNA decay machineries. In the *Drosophila* germline, Aub and Ago3 colocalize with the mRNA degradation proteins Decapping Protein 1/2 (DCP1/2), Maternal expression at 31B (Me31B), and Pacman (PCM) in the nuage, a perinuclear riboprotein assembly, forming a complex (the pi-body) in a piRNA-dependent manner that facilitates mRNA degradation [70] (Fig. 3a). In early *Drosophila* embryos, the key piRNA pathway components Piwi, Aub, Ago3, Armitage (Armi), Spindle-E (Spn-E), and Squash (Squ) are all involved in the deadenylation and decay of maternal *Nanos* (*Nos*) mRNA through a piRNA targeting region in the *nos* 3′ UTR [71]. Aub is known to achieve so by forming a complex with RNA-binding protein Smaug (Smg) and the deadenylase CCR4 at the sites of piRNA–mRNA interaction (Fig. 3b). The deadenylation machinery further recognizes the retrotransposon sequences in the *nos* 3′ UTR and executes deadenylation within 4 h [71]. In mouse spermatogenic cells, the piRNA-induced silencing complex (pi-RISC) contains MIWI and CAF1 (Chromatin Assembly Factor 1), a key catalytic subunit of the CCR4-NOT deadenylase complex [72] (Fig. 3c). Thus, the piRNA mediated mRNA degradation is not only achieved by MIWI slicing of the target mRNAs but also by concurrent deadenylation, and possibly decapping, of mRNAs.

### piRNAs activate the translation of mRNAs

**Fig. 4:**
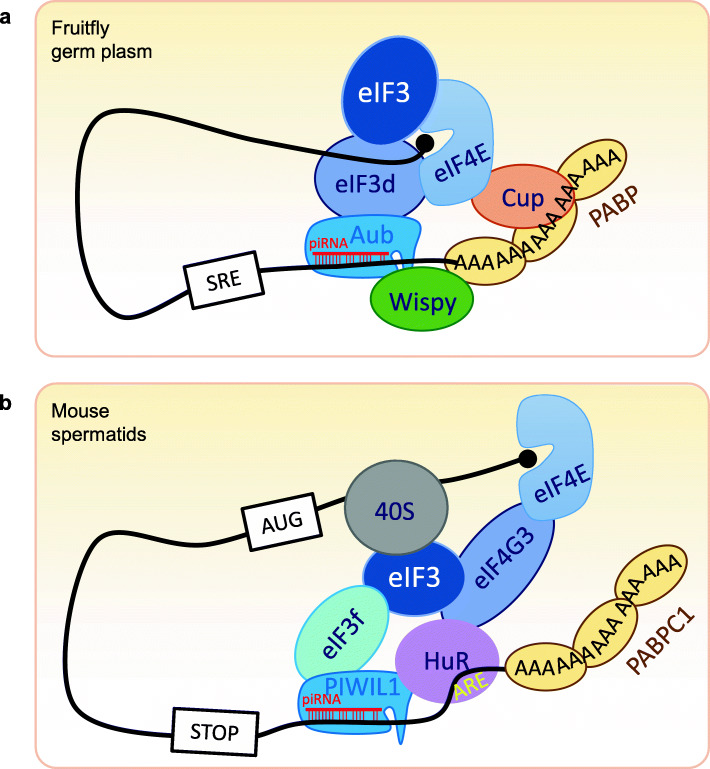
The piRNA-Piwi complex mediates transcriptional activation of target mRNAs. **a** In *Drosophila* germ plasm, Aub interacts with PABP and eIF3 subunits to allow unconventional translation and recruits Wispy poly(A) polymerase to facilitates polyadenylation which also promotes translation [94]. This process is dependent on Cup binding to eIF4E. **b** In mouse spermatids, the piRNA-PIWIL1 complex binds to eIF3f and the 3′ UTRs of ARE-containing target mRNAs, which triggers mRNA looping [95]. HuR binds to the ARE sequences and mediates the association of other translation initiation factors, including eIF4G and PABPC1, to activate translation

The homology between PIWI proteins and eIF2C implies the possibility that they might promote translation, as first proposed for Aub in promoting translation of *nanos* mRNA in *Drosophila* embryos [93]. This function of Aub has been clearly demonstrated by a most recent study [94]. In the *Drosophila* early embryo, Aub physically interacts with eIF3d and the poly(A)-binding protein (PABP) to activate translation of *nanos* mRNA in the germ plasm (Fig. 4a).

PIWI proteins and piRNAs have also been implicated in promoting translation in mammalian systems. MILI is required for germline stem cell self-renewal in the mouse testis [96]. It forms a stable RNA-independent complex with eIF3a and associates with the m7G cap-binding complex that contains eIF4E and eIF4G in spermatogonia and spermatocytes [96]. In 7 dpp spermatogonia (most of which are germline stem cells), the *Mili* mutation has no significant effect on the cellular mRNA level but significantly reduces the rate of protein synthesis. Thus, MILI appears to positively regulate translation for germline stem cell self-renewal.

The MIWI-piRNA complex also associates with the capping complex, including cap-binding protein eIF4E, and regulates translation in the mouse spermatocytes and spermatids [97]. Recently, it has been further reported that, in mouse spermatids, a fraction of piRNAs associate with MIWI and recognize ARE (AU-rich element)-containing target mRNAs through imperfect base pairing in the 3′ UTR of target mRNAs [95]. ARE-binding protein HuR loaded with target mRNA and the translation initiation factor eIF3f form a complex with piRNA and MIWI to further activate the translation of mRNAs required for acrosome formation during spermatid development (Fig. 4b). These lines of evidence illustrate a sophisticated regulatory nexus of PIWI-piRNA complex in modulating the transcriptome at posttranscriptional level. Despite the above findings, the function of PIWI proteins in translational regulation has not been extensively studied and deserves more attention.

### piRNAs regulate the localization of mRNAs

In addition to working with the mRNA decay machinery, the PIWI-piRNA complex has also been reported to be involved in the subcellular localization of mRNAs. For example, in *Drosophila*, the Aub-piRNA complex facilitates the localization of critical germ cell mRNAs to the germ plasm [98]. This function is achieved by working with Wispy, a germline-specific non-canonical poly(A) polymerase. During *Drosophila* oogenesis, Aub participates in the formation of the polar granule that localizes germline-specific mRNAs.

## Regulation of lncRNAs by transposons

Recent studies also indicate that transposon sequences in lncRNAs are the targeting sites of transposon-derived piRNAs that identify these lncRNAs for degradation. Global characterization of transposon distribution in the human genome revealed that the vast majority of lncRNAs (83.4%) overlap with at least one transposon, in contrast to protein-coding sequences—only 6.2% of which overlap with transposons [99]. In the human genome, 75% of lncRNA transcripts contain an exon that originates at least partially from a transposon. Different types of transposons are differentially represented in lncRNA loci, and transposon landscapes differ among various tissue types [99]. LncRNAs that overlap with transposons are less expressed as compared to those that do not overlap with a transposon, indicating a suppressive role of transposon insertion [99].

### Transposons repress lncRNA expression via the PIWI-piRNA pathway

**Fig. 5:**
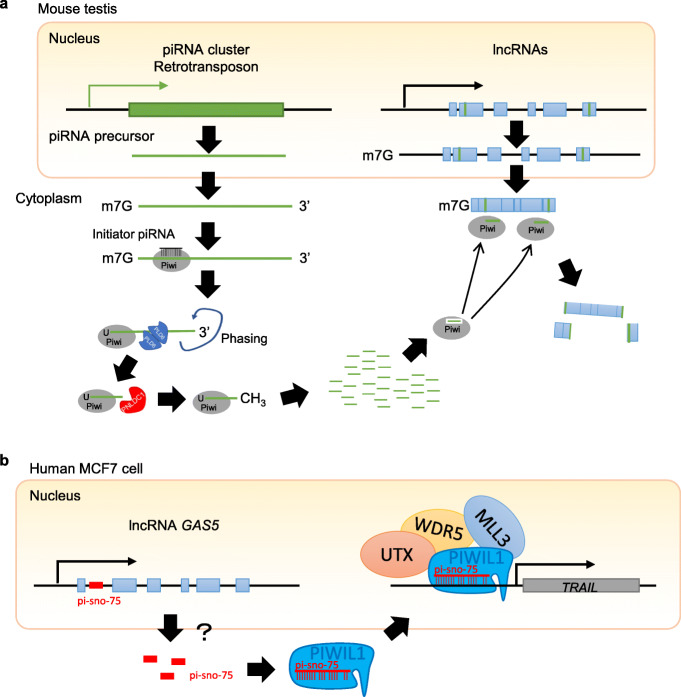
piRNAs mediate transposon regulation of lncRNAs. **a** piRNA mediates post-transcriptional regulation of mRNAs. piRNA precursors are transcribed from piRNA clusters that contain transposon sequences and are processed into mature piRNAs. Mature piRNAs are then loaded onto Piwi proteins and guide the Piwi complex to target lncRNAs that contain complementary transposon sequences to degrade these lncRNAs [68]. **b** piRNA mediates transcriptional regulation of mRNAs. In a human breast cancer cell line (MCF7), the *Growth Arrest Specific 5* (*GAS5*) lncRNA produces piRNAs that are associated with Piwi proteins [100]. This piRNA-Piwi complex then recruits WDR5 and the COMPASS epigenetic complex to the promoter of the *TRAIL* (*transcription of tumor necrosis factor (TNF)-related apoptosis-inducing ligand*) gene to modify its epigenetic status to promote its transcription

The involvement of piRNAs in transposon regulation of lncRNAs has been demonstrated in multiple organisms. In mouse spermatocytes, retrotransposon sequences, mostly SINE, are distributed across the entire length of approximately 1500 lncRNAs that are upregulated in *Miwi*^*−/−*^ testis, which represents ~ 25% of the expressed lncRNAs in these cells [68]. This distribution is in contrast to their predominant localization to the 3′ UTR of mRNAs. The transposon-containing lncRNAs become overexpressed in *Miwi* and *Mov10l1* mutants, implying that the transposon regulation of lncRNA expression is via the piRNA pathway [68] similar to its role in mediating transposon regulation of mRNAs (Fig. 5a). Presumably, the PIWI-piRNA complex slices the target lncRNAs in a way similar to their slicing of mRNAs. However, this remains to be directly demonstrated.

### Other regulatory relationships between transposons, lncRNAs, and piRNAs

The piRNA regulation of lncRNA expression can be manifested in different ways. Some piRNA clusters that are expressed during the pachytene stage originate from testis-specific lncRNAs, indicating that lncRNAs themselves can be precursors of piRNAs [14, 101]. Although not as much is known about the relationship among transposons, lncRNAs, and piRNAs in the primordial germ cells, in their precursor cells in humans, i.e., human embryonic stem cells and induced pluripotent stem cells (iPSCs), the presence of transposon families such as HERVH (human endogenous retrovirus H) elements in lncRNAs is correlated with high expression levels of host lncRNAs, implicating that HERVH sequences positively regulate the expression of lncRNAs in a cell-type-specific manner [99], even though it is not known whether such regulation occurs at the transcriptional or post-transcriptional level. In *Drosophila* OSS and OSC cells, transposon insertions around lncRNA stimulate the expression of corresponding lncRNAs in a Piwi-dependent manner [92], implying a possible role of piRNA in transcriptional regulation of lncRNA expression.

In addition, some lncRNAs can produce piRNAs to promote gene expression at the transcriptional level. In a human breast cancer cell line (MCF7), a lncRNA called *Growth Arrest Specific 5* (*GAS5*) facilitates the transcription of *TRAIL* [*transcription of tumor necrosis factor (TNF)-related apoptosis-inducing ligand*] mRNA. This is through *GAS5*-derived piRNAs that are associated with PIWI proteins [100]. These piRNA-PIWI complexes then recruit WDR5 and the COMPASS complex, a key complex for epigenetic modification, to the promoter of the *TRAIL* gene to modify the epigenetic status of the gene, which facilitates TRAIL transcription. This indicates an important role of PIWI-piRNA complex in epigenetic regulation of gene expressions (Fig. 5b). In *Tetrahymena*, piRNAs that are derived from noncoding RNA loci bind to *Tetrahymena* PIWI protein Twi8p and its target lncRNAs, which leads to a decrease in the level of the targeted lncRNAs [102]. These roles are similar to what has been found in mammalian systems.

## The regulation of mRNAs by pseudogenes

There are 14,000 pseudogenes in the human genome [103]. Analysis of human sperm small RNAs reveals that some piRNA clusters are located within pseudogenes [104]. piRNAs derived from pseudogenes are predicted to target protein-coding cognate genes. In mouse late spermatocytes, at least 14 genes are significantly regulated by their pseudogenes [68]. Thus, pseudogene regulation of mRNAs via piRNAs may be a significant mechanism in the germline that has been largely ignored.

**Fig. 6:**
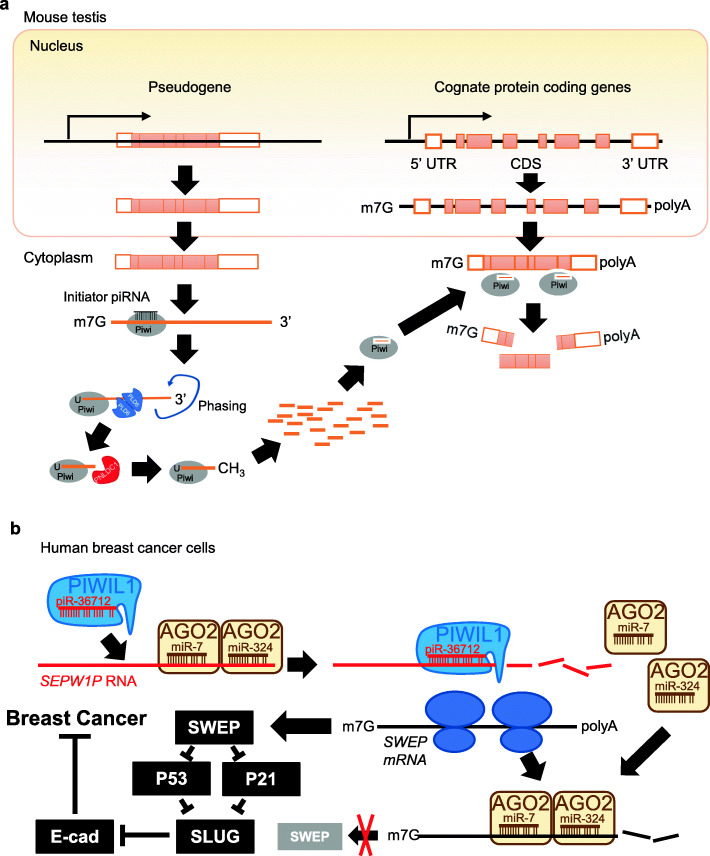
piRNAs mediate pseudogene regulation of cognate mRNAs. **a** Anti-sense transcripts of pseudogenes serve as precursors of piRNAs. These pseudogene-derived piRNAs form Piwi-piRNA complex that binds to complementary sequences in the cognate mRNAs to promote their degradation [68]. **b** In human breast cancer cell lines, the Piwi-piR-36712 complex degrades the RNA of pseudogene *SEPW1P*, which releases *SEPW1P*-RNA-bound miR-7 and miR-324 to bind to cognate *SEPW* mRNA, promoting its degradation and inhibiting its translation [105]. The reduction of SEPW protein weakens its repression towards p53 and p21, allowing p53 and p21 to release Slug suppression towards E cadherin expression, which blocks cancer progression

Recent studies indicate that piRNA derived from pseudogenes can regulate the expression of their cognate mRNAs. The first clear demonstration of such a function comes from a study of the mouse late spermatocyte. The mRNA level of the *Stambp* gene is dramatically increased in *Miwi*^*−/−*^ and *Mov10l1*^*−/−*^ late spermatocytes, indicating that the piRNA pathway may be involved in preventing the over-expression of *Stambp* mRNA [68] (Fig. 6a). However, the *Stambp* mRNA complements to PIWIL1-associated piRNAs that do not map to transposons but uniquely to its pseudogene, *Stambp-ps1* [68]. Indeed, knocking out *Stambp-ps1* expression abolishes the *Stambp-ps1*-derived piRNAs and causes drastic increases in the *Stambp* mRNA level, illustrating the importance of a pseudogene in the regulation of its cognate active gene via the piRNA [68].

Conversely, pseudogenes can also be regulated by piRNAs. In human breast cancer cell lines MCF7 and ZR75–1, a piRNA called piR-36,712 directly binds to pseudogene *SEPW1P* RNA and reduces its level [105] (Fig. 6b). This releases its bound miRNAs miR-7 miR-324, allowing more miR-7 miR-324 to target the mRNA of its cognate gene *SEPW* to reduce its stability and translation*.* The reduced SEWP level in turn weakens its inhibition towards p53 and p21, allowing p53 and p21 to reduce the suppression of E cadherin expression by a zinc finger protein called Slug, which blocks cancer development [105]. This type of regulation may be specific to cancer cells because piRNAs are not detected in normal somatic cells, such as breast epithelial cells,

Pseudogene-derived piRNAs have been identified across the evolutionary tree. In *Tetrahymena*, pseudogene-derived small RNAs have been identified that have binding preference to Twi2 (*Tetrahymena* PIWI2) but not to Twi7 or Twi8 [106]. In the marmoset testis, piRNA clusters contain processed pseudogenes that are antisense-oriented relative to their cognate genes so they can produce piRNAs that target their cognate genes (cf. Fig. 6aa) [107]. In addition, pig piRNA clusters are also highly enriched for exon sequences of pseudogenes [108]. In vertebrate genomes, endogenous bornavirus-like nucleoprotein elements (EBLNs) represent a special type of genomic sequences derived from ancient bornaviral nucleoprotein mRNA via retrotransposition. Human EBLNs are actively transcribed pseudogenes that give rise to piRNAs with a possible role in interfering with ancient bornaviral infection [109]. Thus, regulation by pseudogene-derived piRNAs is likely a conserved regulatory pathway.

## Diverse functions of satellite-repeat derived piRNAs

### Satellite-repeated derived piRNAs negatively regulates mRNA stability

In *Drosophila*, it has been shown that pericentromeric piRNAs, specifically, *AT-chX* piRNAs and *Su(Ste)* piRNAs, also suppresses the *vasa* mRNA of a different species of *Drosophila* during interspecies mating to ensure reproductive isolation [84]. Most recently, it was shown that a large number of piRNAs were derived from a satellite repeat in both the soma tissues and germline of the mosquito *Aedes aegypti* [110]. Two of them, tapiR1 and tapiR2 (tandem repeat-associated piRNA1 and 2), are associated with *Aedes aegypti* PIWI protein Piwi4. The resulting Piwi4-piRNA complex silence target RNAs (both mRNA and lncRNA) via a sequence-specific recognition rule reminiscent of microRNA seed sequence. Notably, the piRNA-generating satellite repeats were highly conserved across mosquito species for approximately 200 million years and are very similar to that of other higher organisms. Hence, there might be an evolutionarily conserved mechanism for post-transcriptional regulation mediated by PIWI-satellite piRNA complex.

### Subtelomeric piRNAs regulate telomeric function

Remarkably, the piRNAs are even involved in regulating telomeres and centromeres. Telomeric and centromeric piRNAs have been reported in various organisms. Shortly after piRNAs were characterized, piRNAs were mapped to subtelomeric regions of the *Drosophila* genome. One of these piRNAs, the 3R-TAS1 piRNA, regulates the epigenetic state of the target subtelomeric sequence [111] and is essential for germline stem cell maintenance [112]. Telomeric piRNAs in *Drosophila* are required for the deposition of Heterochromatin Protein 1 (HP1) and its homolog Rhino as well as epigenetic suppression of telomeric retrotransposon [113, 114]. Loss of piRNA pathway proteins results in the significant upregulation of telomeric retroelement transcripts and downregulation of telomeric piRNAs [115–118]. HP1a, a partner of Piwi protein [49, 111], is required for the generation of piRNAs that map to telomeres and peri-centromeres in the *Drosophila* germline [119]. The nuclear CCR-NOT complex is required for the degradation of telomeric transcripts in a Piwi-dependent manner [120]. In addition, *Aub*, *Armi*, and *Ago3* mutations reduce telomeric piRNAs production and disrupt the binding between telomere and telomere protection complex [117, 121]. All these findings together indicate an essential role of the piRNA pathway in subtelomeric and telomeric function in *Drosophila*.

Interestingly, the prevalence of subtelomeric piRNA clusters is significantly higher in the genomes of *Drosophila* in the wilderness than in standardized laboratory *Drosophila* stocks [122]. Since a well-established telomeric function in *Drosophila* is to suppress telomeric retrotransposons, this difference may reflect another function of telomeric piRNAs in the suppression of telomeric retrotransposons, which might be more needed by flies in the wilderness that are possibly more ridden with retrotransposons.

Because mammalian telomeres are composed of simple repeats instead of telomeric retroelement, the telomeric function of piRNA in *Drosophila* is unlikely to be conserved. However, subtelomeric sequence and structure is highly conserved from *Drosophila* to mammals. Hence, some other important function of the subtelomere, such as repressing and regulating nearby euchromatic gene expression (position effect), might be conserved in higher organisms.

In *Tetrahymena*, telomeric repeat-derived small RNAs selectively bind to Twi10 [106]. In *Caenorhabditis,* perfect telomeric small RNAs are extremely rare among small RNAs that are immunoprecipitated with Ago proteins; however, there are significant numbers of telomeric small RNAs with mismatches were associated with Ago proteins [123]. These small RNAs might be involved in telomere protection in a way similar to that in *Drosophila*.

### Pericentromeric and centromeric piRNAs regulate centromere function

In *Drosophila*, pericentromeric satellite repeats are known to be crucial for centromere function and give rise to piRNAs [13, 28, 124] that are critical for the suppression of *satellite* RNA expression [28, 125, 126]. The involvement of piRNAs in telomeres and centromeres is likely to be crucial for the genome integrity and segregation during germ cell division. A clear requirement of the Piwi-piRNA pathway for chromosome segregation is illustrated by a most recent study in mice, where MIWI prevents aneuploidy during meiosis by piRNA-guided cleavage of excess major and minor satellite RNAs [127]. This allows normal assembly of homologous kinetochore pairs. Overexpression of these satellite RNAs in wildtype meiotic cells also causes aneuploidy. Dicer facilitates the degradation of MIWI cleavage products and other double-strand satellite RNAs. These findings, supported by another latest study on Dicer [128], start to reveal a direct role of PIWI proteins and satellite RNAs in chromosome segregation during meiosis.

## Conclusion

A eukaryotic genome is composed of protein-coding genes, transposons, pseudogenes, centromeres, telomeres, and other repeat sequences. Recent studies, mostly in the germline, start to reveal important roles of piRNAs in functionally linking these major constituents of the genome. piRNAs can be derived from transposon RNAs, pseudogene RNAs, mRNAs, lncRNAs, and telomeric/centromeric repeat RNAs and subsequently partner with PIWI proteins to recognize complementary sequences in these RNAs. These observations together start to reveal a complex network of regulation mediated by piRNAs that unifies the genome at the post-transcriptional level. This is perhaps the first regulatory network of any kind that executes genome-wide regulation in any cell type. Future investigations on this complex network at the mechanistic level will further reveal how this network interacts with other canonical RNA regulation mechanisms to define the genomic function in germ cells and shed light on such function in other cell types.

## Glossary

Transposon: A DNA segment that can undergo transposition into a different site in the genome.
Retrotransposons: Transposons that transcribe itself into a RNA intermediate for reverse transcription to produce double-stranded DNA for transposition (insertion).
Pseudogene: A DNA segment that is an imperfect copy of a functional gene, often including a (sometime altered) promoter. Most pseudogenes are transcribed but not translated due to mutations in protein-coding sequences.
lncRNA: Long non-coding RNAs, which are conventionally defined as RNAs that are at least 200-nucleotide in length but do not contain a protein-coding sequence.
Dicer: A type of RNase III family endoribonucleases that cleave double-stranded RNA into short double-stranded RNA fragments of mostly 21–22 nucleotides.
Centromere: The region of a chromosome, containing highly repetitive DNA sequence, that serves as the attachment site for microtubules of the spindle during cell division.
Pericentromere: The chromosomal regions flanking the centromere that contains highly repetitive DNA and are heterochromatic.
Telomere: The two end-regions of a chromosome that protect the chromosome from shortening after DNA replication or from fusion to other chromosomes.
Subtelomere: The two regions of a chromosome that is right next to the telomere, containing of highly repetitive DNA sequences and are heterochromatic.
Pachytene: A mid-stage of the prophase of meiosis I, following zygotene, characterized by the presence of shortened and thickened paired chromosomes, with the two chromatids clearly separate.
